# Application of patient-derived induced pluripotent stem cells and organoids in inherited retinal diseases

**DOI:** 10.1186/s13287-023-03564-5

**Published:** 2023-11-27

**Authors:** Yuqin Liang, Xihao Sun, Chunwen Duan, Shibo Tang, Jiansu Chen

**Affiliations:** 1 Aier Eye Institute, Changsha, 410015 China; 2 Eye Center of Xiangya Hospital, Central South University, Changsha, 410008 China; 3 Changsha Aier Eye Hospital, Changsha, 410015 China; 4 Key Laboratory for Regenerative Medicine, Ministry of Education, Jinan University, Guangzhou, 510632 China

**Keywords:** Retinal organoid, Induced pluripotent stem cell, Inherited retinal disease, Disease modeling, Tissue engineering

## Abstract

Inherited retinal diseases (IRDs) can induce severe sight-threatening retinal degeneration and impose a considerable economic burden on patients and society, making efforts to cure blindness imperative. Transgenic animals mimicking human genetic diseases have long been used as a primary research tool to decipher the underlying pathogenesis, but there are still some obvious limitations. As an alternative strategy, patient-derived induced pluripotent stem cells (iPSCs), particularly three-dimensional (3D) organoid technology, are considered a promising platform for modeling different forms of IRDs, including retinitis pigmentosa, Leber congenital amaurosis, X-linked recessive retinoschisis, Batten disease, achromatopsia, and best vitelliform macular dystrophy. Here, this paper focuses on the status of patient-derived iPSCs and organoids in IRDs in recent years concerning disease modeling and therapeutic exploration, along with potential challenges for translating laboratory research to clinical application. Finally, the importance of human iPSCs and organoids in combination with emerging technologies such as multi-omics integration analysis, 3D bioprinting, or microfluidic chip platform are highlighted. Patient-derived retinal organoids may be a preferred choice for more accurately uncovering the mechanisms of human retinal diseases and will contribute to clinical practice.

## Background

Inherited retinal diseases (IRDs) affect millions globally and have become one of the leading causes of irreversible vision loss in children and the working population in developed countries [1–3]. IRDs, a group of disorders with high clinical and genetic heterogeneity, are associated with 317 pathogenic genes, among which 281 have been identified (RetNet: http://sph.uth.edu/retnet/, last accessed 10 June 2023). These genes have been found to play roles in almost all aspects of retinal structure and function, including retinal development, phototransduction, visual cycle, ciliary trafficking, ion channels, phagocytosis, mitochondrial function, protein degradation, outer segment structure, and pre-mRNA splicing [4]. Substantial progress has been made in elucidating the molecular genetic factors involved in IRDs and mutation screening techniques in the past two decades [5, 6]. However, the pathological mechanisms associated with specific genotypes still need to be better understood, owing to the availability of limited treatment options. Establishing accurate and available disease models, categorized by mutation and disease phenotype, is vital for gaining insight into IRDs.

Rodents have been widely employed as experimental models for studying the pathogenesis and treatment of human genetic diseases. However, they suffer the demerits of not altogether representing the occurrence and progression of the disease due to the translation barrier between rodents and humans [7, 8], e.g., mice are deficient in a macula on their retinas. Moreover, many studies reported that IRD mice fail to capture the pathological features of retinal photoreceptor degeneration [9–12].

Induced pluripotent stem cells (iPSCs) have the inherent merit of unlimited proliferation, self-renewal capacity, and multidirectional differentiation [13]. Human iPSCs can retain the unique genomic information of each individual since they are derived from autologous cells and devoid of limitations posed by embryonic stem cells, like ethical issues and immune rejection after transplantation [14]. Human iPSCs from somatic cell reprogramming have opened up an entirely new perspective for obtaining patient-specific cell lines, which are then differentiated into desired cell types under appropriate conditions, including retinal ganglion cells, vascular endothelial cells, cardiomyocytes, osteoblasts, hematopoietic cells, and neurons [14, 15]. In recent years, the emergence of three-dimensional (3D) organoids capable of forming complex tissue-like structures has gradually transformed our ability to model human development and disease, drug screening, and cell therapy [16–18]. Herein, this review systematically summarizes the role of patient-derived iPSCs and organoids in IRDs based on previously published studies.

## Application of patient-derived iPSCs in IRDs

Reprogramming technology is considered one of the most important advances in the field of stem cell research and regenerative medicine [14, 19, 20]. Fibroblasts were the first somatic cells to be applied for reprogramming into human and mouse iPSCs [21, 22]. Subsequently, more and more cell types from patients were identified to induce iPSCs, like peripheral blood mononuclear cells (PBMCs), urine cells, and dermal fibroblasts, usually used as cell sources of human iPSCs, as shown in Table 1. In 2011, the first study of IRD patient-derived iPSCs for disease modeling and drug screening was reported by Jin et al. [23].

**Table 1:** Source of reprogrammed iPSCs in IRDs

Type	Pathogenic gene and locus	iPSC source	Gender	Age	Method	References
RP	*PRPF8* c.5792C > T, p.T1931M	UCs	Male	17	Episomal plasmid electroporation	[148]
	*PRPF6 c.2699*G > A, p.R900H	PBMCs	Female	15	Episomal plasmid electroporation	[149]
	*SLC7A14* c.988G > A, p.G330R	PBMCs	Male	6	Episomal plasmid electroporation	[150]
	*CRB1* c.2249G > A, p.G750D and c.2809G > A, p.A937T	PBMCs	Male	22	Episomal plasmid electroporation	[151]
	*CRB1* c.1369C > T, p.R457X and c.2027C > T, p.T676M	PBMCs	Male	10	Lentiviral vectors	[152]
	*RP1* c.2098G > T, p.E700X	DFs	Female	76	Episomal plasmid vectors	[153]
	*RP1* c.2161_2162insC	DFs	Female	67	Retroviral vectors	[23]
	*RP9* c.401A > T, p.H137L	DFs	Male	39	Retroviral vectors	[23]
	*RHO* c.562G > A, p.G188R	DFs	Male	40	Sendai virus	[154]
	*RHO* c.644C > T, p.P215L	DFs	Female	35	Sendai virus	[155]
	*PRPH2* c.946T > G, p.W316G	DFs	Female	67	Retroviral vectors	[23]
	*RPGR* c.1685_1686delAT	UCs	Male	24	Lentiviral vectors	[48]
	*USH2A* c. CGC > CAC, p.R4192H	Keratinocytes	Unknown	62	Sendai virus	[52]
	*USH2A* c.2209C > T, p.R737* and c.8693A > C, p.Y2898S	DFs	Female	63	Sendai virus	[156]
LCA	*NMNAT1* c.709C > T, p.R237C	PBMCs	Female	1	Sendai virus	[157]
	*AIPL1* c.834G > A, p.W278X	UCs	Unknown	3	Episomal plasmid electroporation	[65]
	*AIPL1* c.834G > A, p.W278X and c.466-1G > A, intron	UCs	Twins	2	Episomal plasmid electroporation	[65]
	*AIPL1* c.265T > C, p.C89R	DFs	Female	31	Sendai virus	[158]
	*CRX* c.695delC	DFs	Male	6	Sendai virus	[159]
	*RDH12* c.184C > T, p.R62* and c.437T > A, p.V146D	PBMCs	Male	13	Sendai virus	[160]
	*RDH12* c.619A > G, p.N207D	DFs	Female	40	Episomal plasmid electroporation	[161]
XLRS	*RS1* c.488G > A, p.W163X	PBMCs	Male	16	Sendai virus	[162]
	*RS1* c.527T > A, p.F176Y	PBMCs	Male	13	Episomal plasmid electroporation	[163]
	*RS1* c. 304C > T, p.R102W	UCs	Male	11	Sendai virus	[164]
	*RS1* c.214G > A, p.E72K	PBMCs	Male	8	Episomal plasmid vectors	[165]
	*RS1* c.305G > A, p.R102E	PBMCs	Male	7	Episomal plasmid vectors	[166]

*UCs*—urine cells; *PBMCs*—peripheral blood mononuclear cells; *DFs*—dermal fibroblasts

*Termination codon

Although the human dermal fibroblast extraction and culture are convenient, it requires an invasive sampling of donor skin tissue which might translate into permanent scarring. Moreover, DNA variations within the cells are another potential obstacle due to long-term exposure of skin tissue to ultraviolet rays from sunlight [24]. As an alternative source, PBMCs can be isolated from routine blood samples with written informed patient consent and then reprogrammed into iPSCs, since most of the blood withdrawal methods use peripheral venipuncture techniques, which means less trauma and pain for the donor, but it has been reported that blood samples kept at room temperature for a long time without timely processing translate into a decreased number of iPSC colonies; nevertheless, they can be cryopreserved without affecting their reprogramming efficiency [25]. Compared to fibroblasts and PBMCs, urine cell extraction is non-invasive, convenient, simple, reproducible, and discomfort-free, facilitating the willingness of most participants for autologous urine collection. Urine samples are considered an ideal cell source for reprogramming technology because of their prominent advantages. However, poor proliferation and low success rates of urine cells derived from healthy adults and patients have been reported [26].

The preferred somatic cell source for generating iPSCs still needs more consensus since selecting a suitable cell source depends on the actual situation owing to the difference in extraction, culture, and expansion [27]. Notably, patient-specific iPSC cell source is generally required to be consistent with normal control-derived iPSCs. Furthermore, additional measures need to be taken while sampling to avoid contamination, such as skin surface disinfection, sterile disposable gloves and masks, and timely transportation and extraction of somatic cells. Mycoplasma detection of all cell samples was performed regularly. Uncontaminated and well-conditioned iPSCs are more conducive to differentiation into target cells and organoids.

## Application of patient-derived organoids in IRDs

More and more evidence indicated that patient-derived retinal organoids (ROs) have the potential to serve as an ideal platform for tissue and organ reconstruction and in vitro disease modeling [28–30]. The main reasons are listed as follows: (1) ROs with laminar structure are similar to the natural retina and have a variety of tissue-specific cells, including photoreceptor cells, retinal pigment epithelium (RPE) cells, Müller glial cells, ganglion cells, amacrine cells, and bipolar cells [31]. (2) ROs show high reproducibility and fidelity of retinal development [17, 32, 33]. (3) Human iPSC-derived ROs have the advantages of fewer ethical concerns, easy availability, and large-scale production [34]. An overview of recent advances in patient-derived ROs in IRDs is below and summarized in Table 2.

**Table 2:** Summary of patient-derived ROs and RPE cells in IRDs

Type	Gene	Model	Main results	Reference
RP	*PRPF8* c.6974 6994del	RPE cells	Widespread changes in alternative splicing events and dysregulated expression of genes involved in the splicing process and ribosome	[40]
	*PRPF31* (1) c.1115_1125del11 (2) c.522_527 + 10del (3) c.709_734dup (4) c.269_273del	ROs and RPE cells	RPE functional and ultrastructural abnormalities, progressive photoreceptor degeneration, impaired pre-mRNA splicing, and disrupted splicing in cellular adhesion and cilia genes	[41, 44]
	*RP2* c.358C > T, p.R120X	ROs	Loss of RP2 protein led to photoreceptor cell death and outer nuclear layer thinning	[167]
	*RPGR* c.1415-9A > G	ROs	Mislocalisation of rhodopsin and cone L/M opsin, increased photoreceptor apoptosis, and F-actin dysregulation	[46]
	*USH2A* c.8559-2A > G and c.9127_9129delTCC	ROs	Decreased proliferation and laminin expression Abnormal retinal neuroepithelium differentiation and polarization caused defective retinal progenitor cell development and retinal layer formation and disordered organization of neural retina	[53]
	*IMPG2* p.Y254C and p.A805*	ROs	Lack of an outer segment layer and interphotoreceptor matrix disruption due to loss of IMPG2 protein expression or its inability to undergo normal post-translational modifications	[56]
	*CRB1* (1) c.3122T > C, p.M1041T (2) c.2983G > T, p.E995* and c.1892A > G, p.T631C (3) c.2843G > A, p. C948T and c.3122T > C, p.M1041T	ROs	A moderate loss of photoreceptor nuclei in a row, strongly reduced levels of CRB1 variant protein with unaffected *CRB1* transcript levels, and a dysregulated molecular gene profiling of Müller glial cells and rods	[168]
	*PDE6B* c.694G > A, p.E232K	ROs	Mislocalization of rhodopsin and M-opsin in patient ROs with immature morphology Elevated cGMP levels and significant changes in cGMP hydrolysis-related genes	[169]
	*MERTK* (1) c.225delA and c.370C > T, p.Q124* (2) c.225delA	RPE cells	Defective POS phagocytosis	[170]
LCA	*AIPL1* (1) c.834G > A, p.W278X (2) c.834G > A, p.W278X and c.466-1G > C (3) c.834G > A, p.W278X and c.665 G > A, p.W222X	ROs	Reduced photoreceptor-specific PDE6 and increased cGMP levels	[65]
	*CRX* (1) c.464_465insGGCA (2) c.262A > C, p.K88Q (3) c.264G > T, p.K88N (4) c.413delT	ROs	Defective photoreceptor maturation and diminished visual opsin expression	[66, 67]
	*CEP290* c.2991 + 1655A > G	ROs and RPE cells	Aberrant splicing and impaired ciliogenesis	[68, 171]
	*LCA5* c.835C > T, p.Q279*	ROs	Lack of lebercilin expression and ciliary localization as well as mislocalization of rhodopsin and cone L/M opsin	[70]
	*IQCB1* (1) c.659delC and c.1362C > T, p.R455X (2) c.421_422delTT and c.1036G > T, p.E346X (3) c.1382C > T, p.R461X and c.1516_1517delCA	ROs and RPE cells	Elongated cilia morphology, impaired development of outer segment structures, and mislocalization of visual proteins	[172]
XLRS	*RS1* (1) c.625C > T, p.R209C (2) c.488G > A, p.W163X	ROs	Retinal splitting, defective retinoschisin production, abnormal photoreceptor development, and altered paxillin dynamics	[75]
Batten disease	*CLN3* c.175G > A, p.A59T	ROs	Altered pre-mRNA splicing, accumulation of subunit-C of mitochondrial ATPase, mislocalization of peroxisomes, and vacuolization of photoreceptor inner segments	[76]
Achromatopsia	*ATF6* c.1699T > A, p.Y567N and c.970C > T, p.R324C	ROs	Cone defects, increased endoplasmic reticulum stress, Müller cell activation, disrupted mitochondrial structure, and elevated respiratory chain activity gene expression	[77]
Bestrophinopathy	*BEST1* c.229C > T, p.P77S	RPE cells	Impaired bestrophin channel activity	[79]

### Retinitis pigmentosa (RP)

RP, the most common type of IRD, is characterized by progressive degeneration of RPE cells and photoreceptors with a prevalence of approximately one in 4000 [35]. It initially manifests as night blindness, visual field constriction, and changes in the fundus, eventually leading to irreversible impairment of central vision [36]. The molecular pathogenesis of RP is not fully understood, and there is still no cure or effective treatment to slow down the disease progression [37, 38]. Patient-derived RPE cells and ROs for modeling RP could recapitulate the genotype–phenotype features of the disease. Due to clinical and genetic heterogeneity, different retinal degeneration phenotypes caused by intra-gene variations and the same phenotype caused by mutations in multiple genes could be presented.

Mutations in pre-mRNA processing factors (PRPFs) are the main cause of autosomal dominant RP related to the formation of the U4/U6.U5 tri-snRNP complex, a core spliceosome component [39]. For instance, RPE cells generated from iPSCs of an RP patient carrying the *PRPF8* mutation showed widespread changes in alternative splicing events and dysregulated expression of genes involved in the splicing process and ribosome, indicating loss of spliceosome function [40]. In addition, ROs and RPE models from patient-specific iPSCs with the *PRPF31* mutation showed impaired pre-mRNA splicing process as described by Baskin et al. On the other hand, abnormal photoreceptor and RPE changes were also observed, including cell morphology, cilium structure, apical-basal polarity, and phagocytosis function of the photoreceptor outer segment (POS) [41]. Such an aberrant phenotype was also observed in iPSC-RPE cells from the *PRPF6-*mutated patients [42]. These reports suggested that progressive RPE and photoreceptor degeneration might be attributed to the mis-splicing of genes vital for retinal structure and function. Similarly, cytoplasmic mislocalization of PRPF31 protein in RPE and photoreceptor cells with reduced expression in nuclear localization has been reported lately [43]. Moreover, the effect of *PRPF31* mutation on the spliceosome impaired U4/U6.U5 tri-snRNP assembly and decreased splicing activity [43]. The mutant PRPF31 protein causes photoreceptor cell degeneration in organoids, with rods expiring first, followed by cones, which correspond to the results obtained in RP patients [44]. In contrast, some transgenic mice with *prpf3*^*T494M/*+^, *prpf8*^*H2309P/*+^, and *prpf31*^±^ exhibited unsatisfactory performances in photoreceptor degeneration [9, 45].

The protein trafficking function of connecting cilia in photoreceptors is regulated by the retinitis pigmentosa GTPase regulator (RPGR), which is necessary for photoreceptor development. In the context of disease modeling, patient-derived ROs showed that gelsolin failed to be activated due to disturbed interaction between mutant RPGR protein and gelsolin, resulting in impaired F-actin disassembly of cilia and mislocalization of photoreceptor markers rhodopsin and opsin [46]. Mice with knockout *RPGR* and *Gelsolin* showed significant abnormalities in F-actin polymerization and rhodopsin expression [47]. Therefore, using patient-derived ROs could explain the ciliary phenotype of F-actin dysregulation as a unique RPGR mechanism. To explore the effectiveness of in vitro gene editing, Deng et al. performed CRISPR/Cas9-mediated gene correction of *RPGR* mutation to restore expression levels of target genes and proteins, thereby rescuing ciliary lesions and photoreceptor loss in iPSC-derived organoids from three RP patients [48]. Gene therapy for heterogeneous IRD may be a promising strategy to address the underlying molecular defects, but its development in clinical treatment remains a challenge.

Additionally, early retinal development is impeded in some forms of RP. Mutations in the *USH2A* gene encoding usherin protein induce autosomal recessive non-syndromic RP and Usher syndrome [49]. The usherin has been known to house several motifs associated with extracellular matrix (ECM) proteins, such as laminin and fibronectin type III, which are essential for supporting the centrosome-cilium interface and the inner segment/outer segment region of photoreceptors [50, 51]. The patient-specific ROs model carrying the *USH2A* mutation was established a decade ago [52]. Recently, a study revealed defective retinal progenitor cell development and neuroretinal layer formation due to abnormal retinal differentiation and polarization in the *USH2A-*related ROs, where increased apoptosis was observed in the mutated organoids along with decreased proliferation and laminin expression on day 34 compared to the normal control group [53]. Moreover, multi-omics data analysis showed that the down-regulation of ECM organization promoted patient-derived iPSCs and ROs apoptosis via the PI3K-Akt signaling pathway [37].

The gene *IMPG2* encodes interphotoreceptor matrix proteoglycan 2, a protein expressed by cone and rod photoreceptor cells that plays a role in supporting the growth and maintenance of light-sensitive POS [54]. Mutation in *IMPG2* is associated with a severe form of autosomal recessive RP. *Impg2* knockout mice exhibited a relatively mild and late-onset photoreceptor phenotype compared to human disease [55]. Human iPSC-ROs harboring patient-specific or gene-edited mutations in *IMPG2* universally lacked a functional POS layer and interphotoreceptor matrix disruption due to loss of IMPG2 protein or its inability to undergo normal post-translational modification. This POS phenotype was reversed after the correction of the *IMPG2* mutation by CRISPR/Cas9 gene editing. Interestingly, transplantation of *IMPG2*-mutated ROs into the protected subretinal space of immunodeficient rats restored POS growth, suggesting that POS is vulnerable to mechanical stress environment [56].

### Leber congenital amaurosis (LCA)

LCA is the most severe form of IRD, leading to congenital or early-onset blindness [57, 58]. Patients typically present with nystagmus, poor pupillary light response, severe retinal degeneration, and nearly disappeared full-field electroretinogram in infancy or childhood [59]. Luxturna was approved by the Food and Drug Administration (FDA) in 2017 as the first gene therapy drug in ophthalmology, but it is only available for biallelic *RPE65* mutation-associated LCA with a mounting cost of $850,000 [60]. To date, at least 26 pathogenic genes have been linked to LCA, mainly in an autosomal recessive inheritance pattern (RetNet).

Mutation in the aryl hydrocarbon receptor-interacting protein-like 1 (AIPL1) gene is one of the most clinically severe forms of the disease (known as LCA-4 type), accounting for 5%–10% of all LCA cases [57]. AIPL1 protein acts as a photoreceptor-specific cochaperone, interacting with the molecular chaperone heat shock protein 90 (HSP90) to regulate the stability and assembly of phosphodiesterase 6 (PDE6) holoenzyme in the phototransduction cascade, which is responsible for regulating intracellular levels of cyclic guanosine monophosphate (cGMP) in rods and cones [61]. The phenotype of patient iPSC-derived ROs in vitro modeling LCA was similar to LCA-4 rodents as described previously [62]. The loss of AIPL1 protein hindered the PDE6 holoenzyme formation, resulting in increased cGMP levels in photoreceptor cells [63, 64]. Similarly, Leung et al. confirmed these molecular pathological changes in AIPL1-mutated ROs derived from four patients and attempted to investigate the effectiveness of the reagent PTC124, a translational read-through-inducing drug [65]. The results showed a slight increase in full-length AIPL1 protein but failed to completely restore the functional expression of PDE6 and reduce the cGMP levels in photoreceptor cells. However, CRISPR/Cas9-mediated gene editing could rescue the mutant phenotype, as observed in the *AIPL1*-corrected organoids [65].

In addition, modeling *CRX* mutation-related LCA using patient iPSC-ROs technique revealed immature photoreceptor cell development and reduced visual opsin expression [66], which were alleviated using *CRX* gene augmentation therapy mediated by adeno-associated virus (AAV) vectors [67]. The organoid model of the cilia gene *CEP290* recapitulated the LCA-10 disease phenotype and exhibited abnormal splicing and ciliary defects [68]. Contrarily, eupatilin, a bioactive flavonoid, has improved cilium formation and length in *CEP290*-associated ROs [69]. CRISPR/Cas9-mediated gene correction of a nonsense variant in *LCA5* rescued lebercilin expression and localization along the ciliary axoneme in patient-derived ROs [70].

### X-linked recessive retinoschisis (XLRS)

XLRS, also called *RS1*-associated IRD, is characterized by a splitting of the neurosensory retina and cystic macular dystrophy affecting the young male population [71]. Retinoschisin encoded by the *RS1* gene is assembled by retinal bipolar cells and photoreceptors, followed by its secretion into the extracellular surfaces as a homo-octameric complex [71]. The protein contains an amino-terminal signal peptide, the RS1 domain, and a discoidin domain, a specialized domain found in a family of extracellular surface proteins that plays an important role in retinal cell adhesion and cell–cell interactions [72]. At present, several XLRS mouse models have been constructed to recapitulate the retinoschisis phenotype [73, 74]. A recent study has shown that this specific retinopathy may occur in patient iPSC-derived ROs [75]. PBMCs were extracted from blood samples of two patients diagnosed with XLRS, reprogrammed into iPSCs, and then induced into RO disease models. On day 150 of differentiation, *RS1* mutant ROs exhibited cyst/schisis-like features similar to the fundus characteristics of retinal splitting between the inner and outer nuclear layers in XLRS patients and mice. Western blotting and immunofluorescence staining showed that patient-derived ROs had aberrant RS1 protein expression and secretion, resulting in altered paxillin dynamics, photoreceptor development, and retinopathy-related gene expression [75]. Subsequently, CRISPR/Cas9-mediated correction of RS1 deficiency effectively reversed pathological changes in morphological structure and molecular expression, and likewise, introducing this *RS1*-specific mutation into normal control iPSCs successfully reproduced the disease phenotypes [75].

### Other IRDs

Other relatively uncommon types of IRDs have also been studied, such as Batten disease, achromatopsia, and best vitelliform macular dystrophy (BVMD). It has been reported that patient-derived RO models of Batten disease with the *CLN3* mutation exhibited altered pre-mRNA splicing, accumulation of mitochondrial ATPase subunit-C, peroxisomes mislocalization, and vacuolization of photoreceptor inner segments [76]. Achromatopsia is characterized by loss of cone photoreceptor function. At the same time, achromatopsia ROs from patients carrying the *ATF6* variants exhibited molecular and cellular phenotypes, including cone defects, increased endoplasmic reticulum stress, Müller cell activation, disrupted mitochondrial structure, and elevated mitochondrial respiratory chain activity gene expression [77]. Intervention with AA147, a lead small molecular ATF6 agonist, may enhance cone photoreceptor growth and gene expression in the disease ROs by promoting Class 1 *ATF6*-regulated transcriptional activity [78]. In addition, impaired bestrophin channel activity was observed in BVMD patient-derived RPE cells with the *BEST1* mutation, which was restored by AAV-mediated *BEST1* gene augmentation [79, 80].

Taken together, a genotype–phenotype correlation of the disease was corroborated through a series of tests and analysis in patient iPSC-derived RO models, which can accurately reflect instead of mimic the complex clinical and genetic background of human retinal disease, may provide a very favorable experimental tool and platform for launching relevant research, and may also contribute to future drug development and gene therapy strategies. Recently, a clinical trial of a CRISPR/Cas9-mediated gene therapy drug for RP disease was conducted in China (NCT05805007).

## Preclinical application

In recent years, stem cell-derived ROs can be prepared into suitable retinal sheets or purified photoreceptor cells for transplantation in animal models of retinal degeneration to restore the structural and functional integrity of the host retina [81–85]. Notably, purified photoreceptors can directly form host-graft synaptic contact but seldom survive for long after transplantation [86]. In contrast, neuroretina-like graft sheets develop a structured layer in the form of a rosette that promotes graft photoreceptor survival and synaptic interaction with host bipolar cells, and retinal ganglion cell responses to light can be detected via multiple electrode arrays in end-stage retinal degeneration models [87, 88]. A protocol for the preparation, quality control, and transplantation of retinal sheets into retinal degeneration rats has been established and validated previously [82]. Based on prior proof-of-concept studies, Kobe City Eye Hospital has launched the first human clinical trial in Japan using retinal sheets from allogeneic iPSC-derived ROs for transplantation in advanced RP patients (jRCTa050200027). Two patients underwent the surgery, and no serious adverse events have been reported for at least one year following transplantation. Since the graft sheet with a tiny area of approximately 0.5 × 1 mm was delivered into a limited area, improving visual function may be insufficient and requires a better version to induce adequate efficacy [89]. Furthermore, the presence of bipolar cells and their established synaptic connections within the graft may impede graft-host neural integration. Yamasaki et al. induced the *ISL1* gene deletion to significantly reduce the number of retinal bipolar cells to enhance functional integration after transplantation [90]. Besides RO transplantation therapy, a clinical trial of intestinal organoid transplantation in patients with ulcerative colitis was approved in February 2020 and conducted at Tokyo Medical and Dental University in Japan (jRCTb032190207).

## Challenges in the clinical application of iPSCs and organoids

With the rapid progress of regenerative medicine and precision therapy, human iPSCs and 3D organoids play a prominent role in cell transplantation, gene therapy, and drug testing [29, 91–93]. Treatment decisions will become multi-faceted and personalized. Using human iPSC derivatives for transplantation is a way to avoid or reduce the risk of autoimmune rejection, as these cells can be derived from patient samples [94, 95]. However, the clinical application of human iPSCs and organoids is contentious, related to the tumorigenicity and heterogeneity of iPSCs, as well as the absence of standardized culture protocols, such as viability and batch effect of iPSC-derived cells or organoids.

### Regulatory requirement

The development of iPSCs and organoids is an important step in overcoming a clinical application challenge, which requires the use of bioprocesses that are compliant with quality and regulatory guidelines. Regulatory affairs for new cellular products may vary globally, but they are usually manufactured under current good manufacturing practice (GMP) conditions [96]. The establishment of automated and high-throughput methods, supported by machine learning and advanced robotics, will contribute to product consistency, repeatability, and traceability in future clinical applications [97]. Bohrer et al. recently developed a robotic cell culture platform called Cell X to produce clinical-grade patient-specific iPSCs and ROs [98]. Briefly, iPSC clone generation, picking, expansion, and spontaneous retinal formation were all tasks performed by the robotic system, and single-cell RNA sequencing showed that these organoids generated automatically are comparable to those obtained manually [98]. The incorporation of the Cell X robotic platform into iPSCs production and differentiation enables fine labor and time under GMP standards and reduces batch-to-batch variability caused by human error or protocol drift.

In addition, most methods of iPSC generation and retinal induction rely on animal-derived components (i.e., fetal bovine serum) and/or animal-derived matrix molecules or feeder cells [34, 99–101]. However, it is undesirable for cell therapy developers and regulatory agencies to expose clinical-grade cells or organoids to products of animal origin. Recently, Slembrouck-Brec et al. described a defined xeno-free and feeder-free culture condition for the generation of human iPSC-derived ROs and RPE cells [102]. In our previous study, fetal bovine serum was replaced with human platelet lysates to establish a xeno-free ROs culture workflow that facilitates clinical application [103].

### Tumorigenicity

The clinical application of human iPSCs and their derivatives raises issues about efficacy and safety. Mandai et al. reported a clinical study of iPSC-derived autologous RPE cell sheets in two patients with advanced neovascular age-related macular degeneration [104]. The first patient underwent surgical removal of the neovascular membrane followed by subretinal transplantation of an autogenous iPSC-derived RPE cell sheet. One year after surgery, there was no sign of graft rejection or recurrence of the neovascular membrane. However, three abnormal DNA copy number mutations were detected in RPE cells from another patient; therefore, surgery was not conducted because it might affect gene expression dysregulation [104]. Many researchers have found that either iPSC-derived grafts may retain undifferentiated stem cells or immature progenitor cells that continue to proliferate [105, 106], or genetic mutations during in vitro culture may drive tumorigenesis [107]. Moreover, if transcription factors used in reprogramming technology are integrated into the cell genome, specifically the c-Myc factor, teratomas or tumors may emerge [108]. However, only some studies have focused on the genetic safety of iPSC-derived autografts; thus, their long-term in vivo safety still needs to be well understood.

### Heterogeneity

The iPSCs heterogeneity in differentiating potential is a hurdle for downstream applications, including drug screening, gene therapy, and cell therapy. Human iPSCs and their derivatives vary in efficiency across cell lines, which may be attributed to genetic background, epigenetic variables, passage, and culture protocols. For example, most normal iPSCs induce ROs effectively, but a few indeed exhibit inefficiencies or cannot generate retinal tissues [109]. Recently, an optimized system with the addition of recombinant Dickkopf-related protein 1 (DKK1) could significantly improve ROs' self-organization capacity in specific iPSC lines [110]. Additionally, genetic abnormalities affect the development of patient-derived ROs with distinct diseases or variants. Mahato et al. reported that the retinal forming efficiency of RP disease-specific iPSCs was identical to that of the healthy control cells; however, iPSCs with the *RB1*^*−/−*^ mutation failed to form eye field primordial structures [111]. The passage approach employing enzyme and manual purification was more effective than flow cytometry-based sorting for high-yield purification of functional RPE cells from diverse stem cell sources [112]. Although many protocols for generating iPSC-derived ROs have been developed, there are still differences among them. Given this, if iPSCs and their derivatives are to be used clinically, conditions for iPSCs culture and differentiation must be standardized, and regular monitoring of genetic variation throughout the process must be emphasized. In addition, rigorously designed preclinical studies in large animal models are required. Assessing the long-term efficacy and safety of iPSC-based therapies will be meaningful to promote clinical applications in future.

## Future trends

**Fig. 1:**
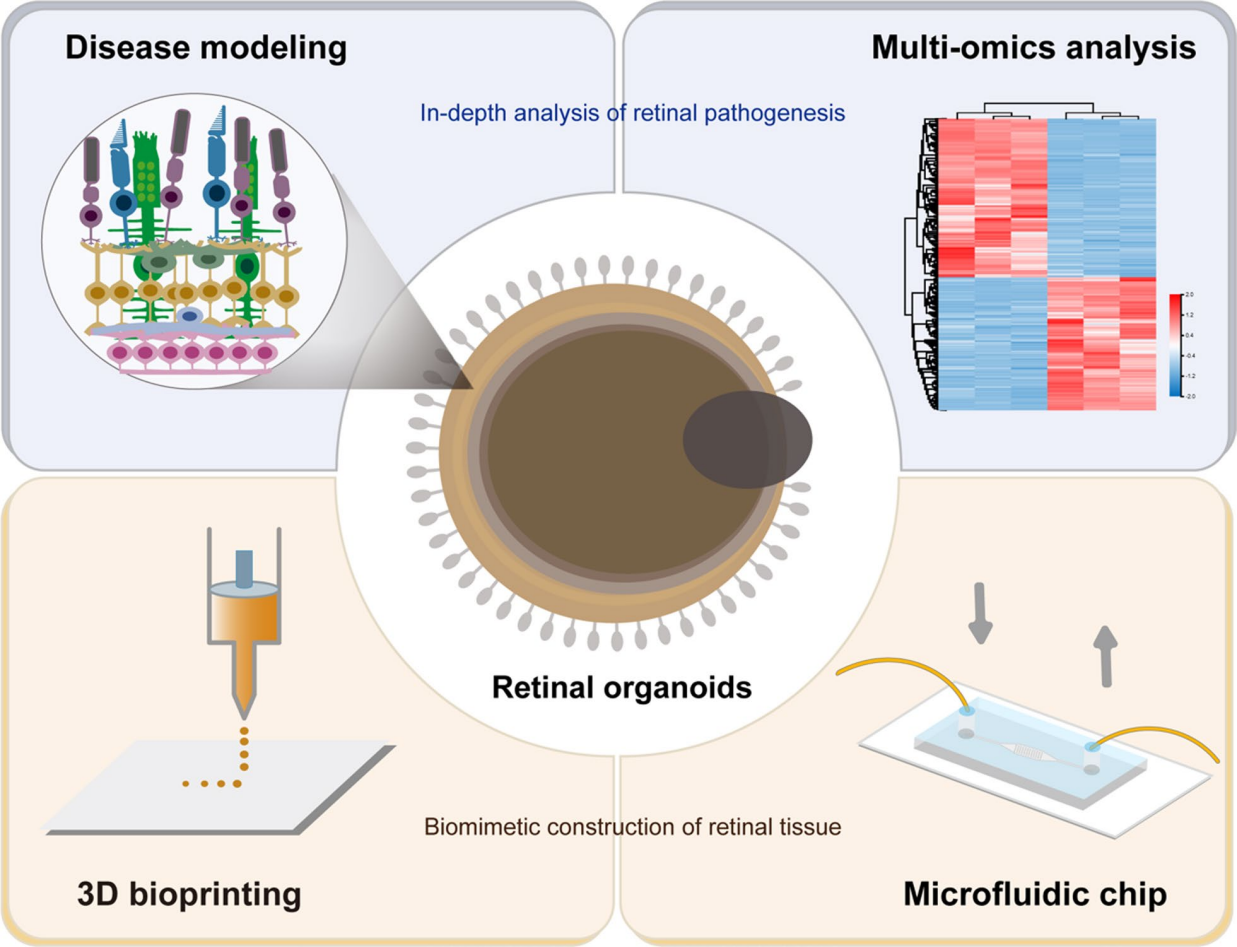
Future trends in the application of patient iPSC-derived ROs. Human organoid technology can be used for disease modeling, in-depth analysis of retinal pathogenesis in combination with multi-omics data, or biomimetic construction of retinal tissue in combination with 3D bioprinting and microfluidic chips

Patient-derived ROs have been used to model IRDs, enabling the recapitulation of disease genotype–phenotype features in vitro. Recently, human organoid technology has integrated multi-omics data to deeply analyze the pathogenesis of retinal diseases, or combined with microfluidic chip platform and 3D bioprinting technology to create more mature and complex organoids [113], which may become the development direction in disease research and tissue engineering (Fig. 1).

### Multi-omics integration analysis

Although IRDs occur due to mutations in the causative gene, the exact molecular mechanisms remain unclear, and more effective treatment strategies are to be discovered [114, 115]. With advances in high-throughput sequencing technology, genomics, epigenomics, transcriptomics, proteomics, metabolomics, and single cell-omics are frequently used in research to better understand biological processes at the gene, protein, and metabolic levels and discover new biomarkers and therapeutic targets [116]. However, single omics data is insufficient for studying systems biology across multiple levels. Multi-omics analysis, which integrates data from two or more omics, has recently been popular in uncovering mechanistic insights [117–120].

It was observed that the *USH2A* mutation dysregulated ECM-related gene expression in patient-derived ROs, which was well validated at transcriptomic and proteomic levels, suggesting an interaction between gene expression and protein synthesis in *USH2A*-related ROs [37]. The degeneration of photoreceptor cells is the main hallmark of IRDs, although the early molecular and cellular events before photoreceptor death are not fully understood. An integrative multi-omics approach was performed in the *Pde6b*^*rd1/rd1*^ mouse model of RP, including temporal transcriptomics of purified rod photoreceptors along with proteomic and metabolomic analysis of the retina [121]. They found that mitochondrial damage and metabolic disruptions are early pathological factors of photoreceptor cell death in retinal degeneration. It was demonstrated for the first time that calcium signaling defects are drivers of mitochondrial and metabolic changes. The molecular mechanisms underlying the onset and early progression of an XLRS mouse model were investigated by combined transcriptomic-proteomic analysis [122]. However, bulk RNA sequencing cannot provide cell-type-specific changes in gene expression. In contrast, single-cell RNA sequencing enables extensive molecular characterization at single-cell resolution and removes the interference caused by diverse cell compositions, making the information obtained more comprehensive [31, 123]. Lee et al. analyzed bulk RNA sequencing data from achromatopsia patient-derived ROs carrying the *ATF6* mutation and identified disrupted mitochondrial structure and abnormal respiratory chain activity gene expression [77]. Single-cell RNA sequencing subsequently indicated considerable down-regulation of cone-related and up-regulation of Müller cell-related genes. Thus, the combination of bulk and single-cell RNA sequencing allows us to establish an integrated understanding of transcriptomes in studying human retinal diseases.

Furthermore, studies based on a multi-omics strategy may help identify biomarkers for early diagnosis or potential therapeutic targets. A recent study showed that microRNA-143 expression was significantly downregulated in oxygen-induced retinopathy rats, and intravitreal injection of its mimics inhibited retinal neovascularization [124]. This is possible by regulating endothelial cell–matrix adhesion and mediating the hypoxia-inducible factor-1 signaling pathway; therefore, microRNA-143 can be used as a potential biomarker and therapeutic target. In addition to attenuating retinal angiogenesis, microRNA-143 had a suppressive effect on retinoblastoma [125]. Bioinformatics analysis of multi-omics data also identified *TTK*, *RRM2*, and *CDK1* as potential retinoblastoma molecular biomarkers [126]. *TTK*, described as an oncogene that promotes tumor progression, was highly expressed in various cancers [127–129], making it a promising therapeutic target.

### 3D bioprinting technology

3D bioprinting technology is the inclusion of 3D printing into tissue engineering and regenerative medicine applications, allowing the rapid and reproducible fabrication of complex biomimetic tissues or organs in vitro, such as 3D-bioprinted ventricles, corneal stroma, skin, bone, and cartilage tissue [130–133]. In recent years, the efficacy of 3D tissue or organ structure printing has been markedly improved due to the rapid development of functional bio-inks.

**Fig. 2:**
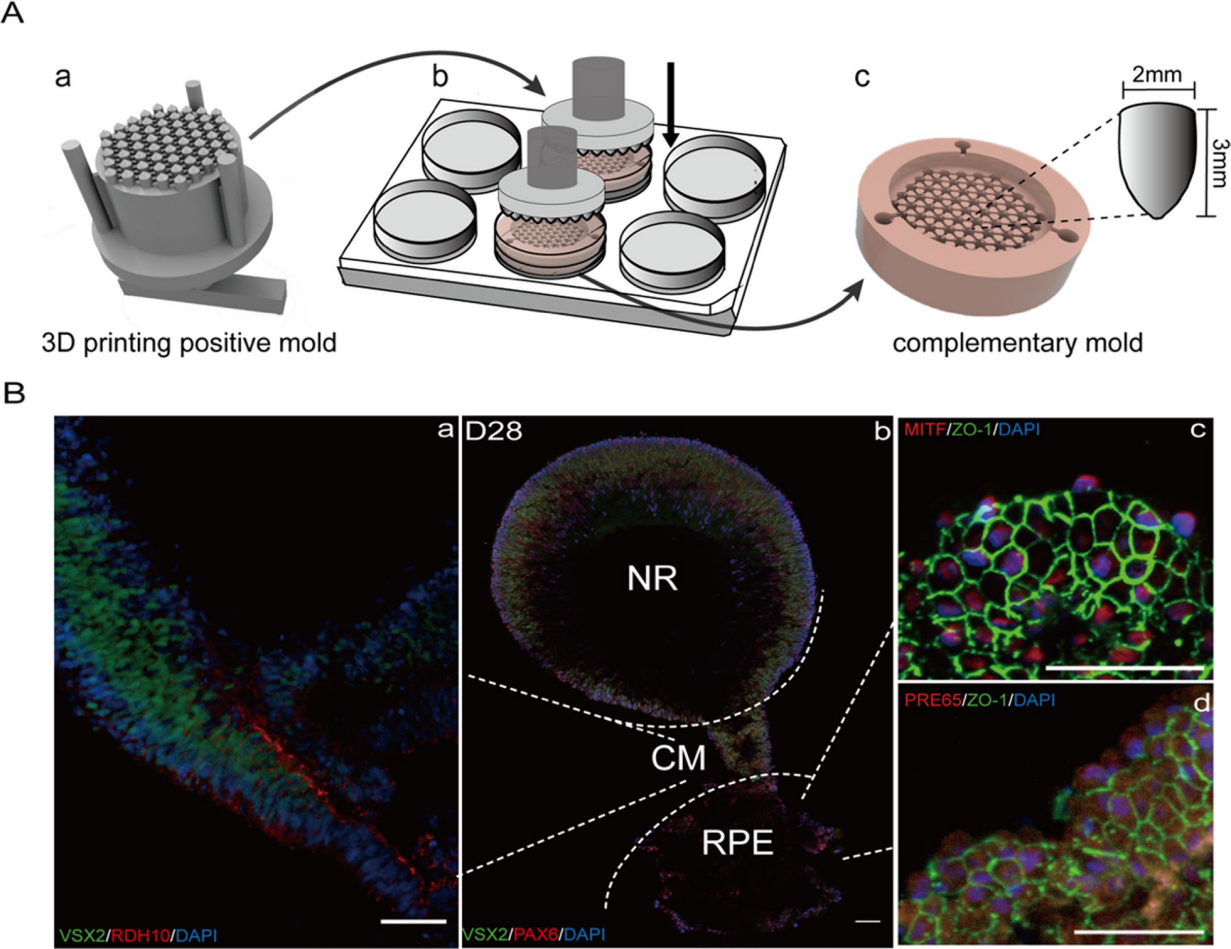
Self-organization of ROs from human iPSCs on a PDMS microwell platform. **A** Schematic diagram of manufacturing PDMS microwell molds, including (a) design and fabrication of 3D-printed positive molds, (b) addition of PDMS biomaterials, and (c) fabrication of complementary PDMS molds. **B** Immunofluorescence staining images of adherent 3D ROs. (a) Ciliary margin domain was stained with RDH10 (red). (b) Neural retina domain was stained with VSX2 (green) and eye field was stained with PAX6 (red). (c, d) RPE domain was stained with ZO-1 (green), MITF (red), and PRE65 (red). Nuclei were labeled with DAPI (blue). Scale bar: 50 µm. [103] Copyright Sun et al. 2023, Biofabrication

Additionally, 3D bioprinting technology is also used for personalized modeling engineering to flexibly design the external shape and internal structure of an object. Despite advances in self-organizing retinal morphogenesis, patient-derived ROs are not currently optimal for testing candidate drugs or cell therapies. For example, ROs often vary in size and quality, contain some off-target tissues and their development may be inconsistent [82]. We previously used 3D-printed polydimethylsiloxane (PDMS) microwell platform for adherent ROs cultivation [103]. Unlike suspended ROs on ultralow adhesion microwell plates, iPSC-derived ROs on PDMS molds were confined to their respective microcavities but shared the same medium and microenvironment, which could not only avoid the fusion of multiple ROs but also ensure the long-term culture and survival of ROs, resulting in efficient and homogeneous ROs with fewer apoptotic cells (Fig. 2). The PDMS microwell platform using 3D bioprinting is envisaged to improve the robustness of in vitro retinal organogenesis and standardization of ROs. However, guiding the proper spatial arrangement of photoreceptor cells for transplantation remains challenging. In 3D scaffolds, retinal progenitor cells harvested from dissociated ROs formed neuronal processes that extended into and aligned with scaffold vertical pores [134]. To precisely establish tissue structures in vitro, strategies based on biomaterials similar to the extracellular microenvironment have been developed to enhance cell characterization. Shrestha et al. used two-photon polymerization to construct a hyaluronic acid (HA) and gelatin scaffold, enabling ECM-derived molecules to offer cellular support and retain significant vitality and proliferation of rat retinal cells [135]. Furthermore, an immersion bioprinting method produced patient-derived brain tumor organoids using HA and collagen bio-inks, where organoids embedded in the HA bath displayed homogeneous volume and geometry for subsequent anti-cancer drug studies [136].

### Microfluidic chip platform

**Fig. 3:**
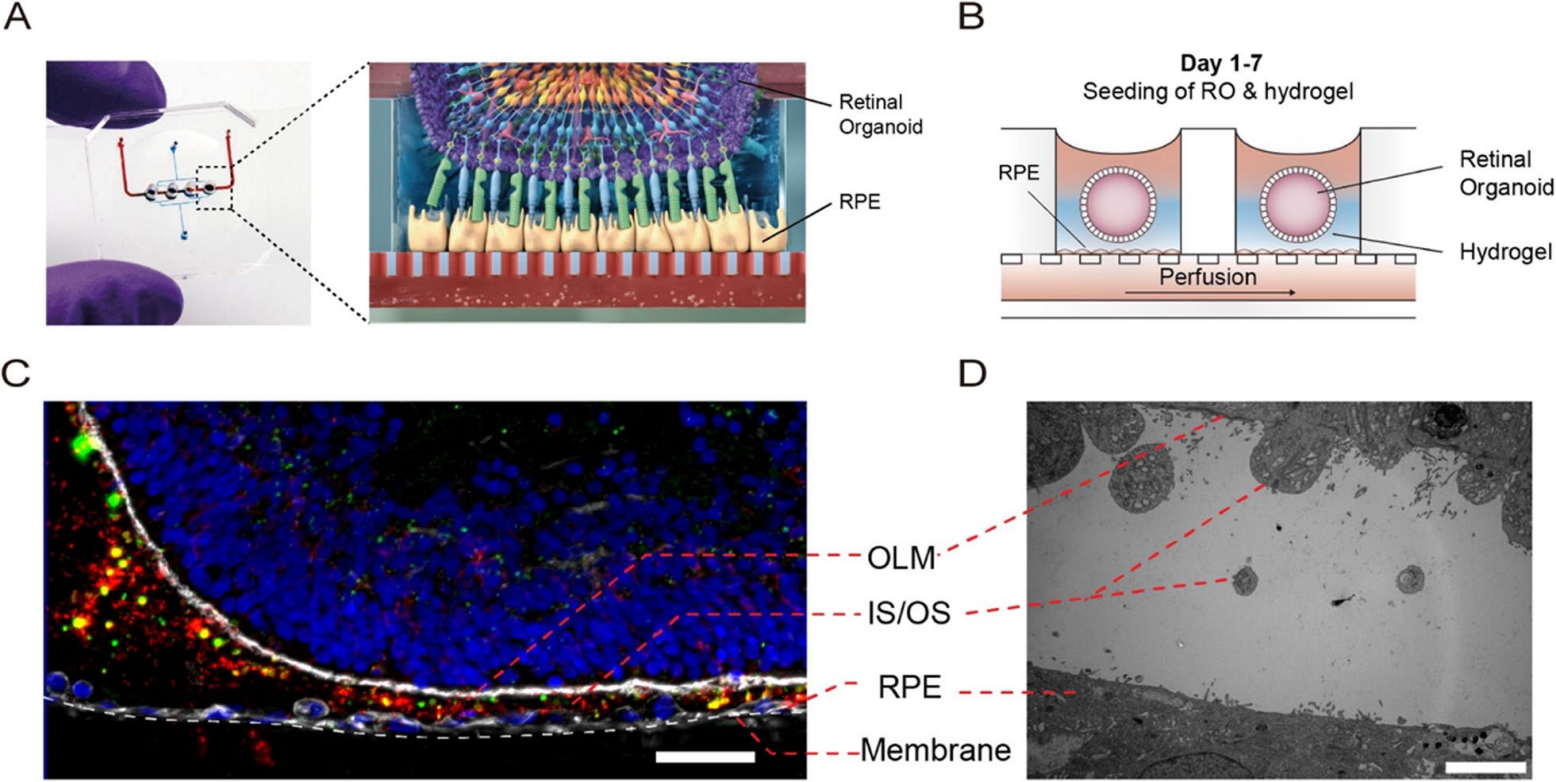
Microfluidic retina-on-a-chip. **A** Photo and **B** schematic representation of ROs and RPE co-cultured in a microfluidic retina-on-a-chip model. **C** Immunofluorescence staining of ROM1 (green), phalloidin (white), and rhodopsin (red) was performed after 7 days of co-culture. Scale bar: 40 µm. **D** Electron microscope image. Scale bar: 5 µm. [29] Copyright Achberger et al. 2019, eLife

Organ-on-a-chip, such as microfluidic retina-on-a-chip models, is an emerging technology that allows the development of novel platforms to simulate the complex structure and microenvironment of the retina in artificially controlled perfusion devices. Briefly, organ-on-a-chip includes the different cell types, structural organization, and microenvironment, usually separated by microporous membranes, offering the advantage of controlling cellular and multiorgan interactions exposed to cultural conditions. Multiple organ-on-a-chip systems that model the interface between RPE and photoreceptor cells, microvascular endothelium and RPE, microglia, and cerebral organoids have been described [29, 137–139]. For example, a study utilized a microfluidic chip platform to co-culture iPSC-derived RPE cells and ROs, which generated the desired pattern, i.e., an outer retinal morphology with vasculature-like perfusion [29] (Fig. 3). After a week of running the microfluidic retina-on-a-chip, it was revealed that photoreceptor calcium dynamics and digested outer segment-like structure signs, replicating retinal basic activities associated with the visual cycle. It was then evaluated with chloroquine and gentamicin, known to induce retinal damage, and the results revealed cell dysfunction and death [29]. Researchers recently analyzed the efficacy, kinetics, and cell tropism of seven different AAV vectors using the retina-on-a-chip platform with satisfactory results [140]. First, they evaluated the performance of different types of AAV vectors in mouse retinas and human iPSC-ROs. Significantly higher fluorescence expression was detected when delivered with the AAV2.7m8 vector, which is consistent with data reported by Dalkara et al. from AAV2.7m8 with highly efficient transduction in the retina of mice and non-human primates [141]. Subsequently, the same vector panel was applied to the retina-on-a-chip model, and the results showed that the AAV2.7m8 vector had stronger transduction signals and cell tropism compared to other AAV types. In addition, two recently developed second-generation AAV vectors, AAV2.NN and AAV2.GL, were analyzed using the retina-on-a-chip platform and subsequently demonstrated their efficient transduction for rod and cone photoreceptors as well as Müller cells [140]. Many retinal diseases involve the outer layers of the retina, including RPE and photoreceptor cell layers. Thus, we hypothesize that IRD patient-specific retina-on-a-chip can replicate the corresponding physiological tissue or organ microenvironment in vitro and has great potential as a tool for high-throughput pharmacology and drug screening.

In addition to modeling the outer retina, two microfluidic organ-on-a-chip models of the outer blood-retinal barrier were reported [137, 142]. In one of the models, RPE and human umbilical vein endothelial cells were co-cultured in a microfluidic chip with microchannels and an open-top culture chamber separated by a polyester membrane [142]. Upon inducing oxidative stress by treating with hydrogen peroxide, a dose-dependent increase in barrier permeability was observed by using a dynamic assay for fluorescence tracing, analogous to the clinically used fluorescence angiography. This method allows semi-quantitative evaluation of the endothelial barrier by analyzing the slope of the fluorescence increase in the perfusion phase and qualitative assessment of lesions and defects by analyzing local fluorescent dye accumulation in the removal phase. They also found that optical coherence tomography could detect changes in microvessel diameter and quality after imaging 3D vascular structures generated by cells in the collagen I hydrogel chip. Another designed model consisted of an iPSC-derived RPE monolayer in the upper compartment and primary human retinal microvascular endothelial cells and choroidal fibroblasts in a hydrogel scaffold in the lower compartment, respectively [137]. After seven days, retinal endothelial cells' vasculogenic self-assembly developed into a dense network of microvessels approximately 10 – 25 μm in diameter, enhancing the RPE phenotype, including intercellular tight junctions, laminin production and deposition, RPE pigmentation, and RPE65 protein expression.

## Conclusions

Human iPSCs and 3D organoid technology play a role in studying human organogenesis and development, disease modeling, drug screening, and preclinical therapies. Recently, the first human clinical trial using intestinal organoids to treat ulcerative colitis patients is ongoing in Japan (jRCTb032190207). In this paper, we reviewed the concept and sources of iPSCs, the recent research advancement of patient-derived iPSCs and organoids in IRDs, and the main challenges that need to be overcome in clinical application. Moreover, multi-omics integration analysis, 3D bioprinting technology, and microfluidic chip platform are further promising patient-derived ROs research avenues.

However, the lack of vascular networks, immune cells, continuous RPE monolayer, and the central nervous system may limit RO generation and development. Co-culture systems for interaction between multi-organoids, or organoids with cells/spheroids, have been studied to address the constraints of traditional organoid cultivation [143–147]. Meanwhile, establishing an organoid culture system with standardized, high-throughput, and undifferentiated operations is required. Homogeneous organoids will simulate the complex organ structure and function, reproduce cell-to-cell communication and molecular features, and explore disease pathogenesis and treatment. Organoids from healthy individuals or patients can also provide a comprehensive evaluation of susceptibility across age, gender, and ethnicity, potentially facilitating the implementation of personalized intervention strategies.

## Data Availability

Not applicable.

## Abbreviations

3D: Three-dimension
AAV: Adeno-associated virus
AIPL1: Aryl hydrocarbon receptor-interacting protein-like 1
ATF6: Activating transcription factor 6
BVMD: Best vitelliform macular dystrophy
Cas9: CRISPR-associated protein 9
CEP290: Centrosomal protein 290
cGMP: Cyclic guanosine monophosphate
CRISPR: Clustered regularly interspaced short palindromic repeats
CRX: Cone-rod homeobox
DFs: Dermal fibroblasts
DKK1: Dickkopf-related protein 1
ECM: Extracellular matrix
FDA: Food and drug administration
GMP: Good manufacturing practice
HA: Hyaluronic acid
HSP90: Heat shock protein 90
IMPG2: Interphotoreceptor matrix proteoglycan 2
iPSCs: Induced pluripotent stem cells
IRDs: Inherited retinal diseases
LCA: Leber congenital amaurosis
PBMCs: Peripheral blood mononuclear cells
PDE6: Phosphodiesterase 6
PDMS: Polydimethylsiloxane
POS: Photoreceptor outer segment
PRPFs: Pre-mRNA processing factors
ROs: Retinal organoids
RP: Retinitis pigmentosa
RPE: Retinal pigment epithelium
RPGR: Retinitis pigmentosa GTPase regulator
RS1: Retinoschisin 1
snRNP: Small nuclear ribonucleoprotein particle
UCs: Urine cells
USH2A: Usherin
XLRS: X-linked recessive retinoschisis
